# The South African Rugby Union: SA Rugby Injury and Illness Surveillance and Prevention Project (SARIISPP): The Currie Cup Premiership Competition, Injury Surveillance Report, 2018

**DOI:** 10.17159/2078-516X/2018/v31i1a6532

**Published:** 2019-01-01

[Fig f21-2078-516x-31-v31i1a6532]

**Figure f21-2078-516x-31-v31i1a6532:**
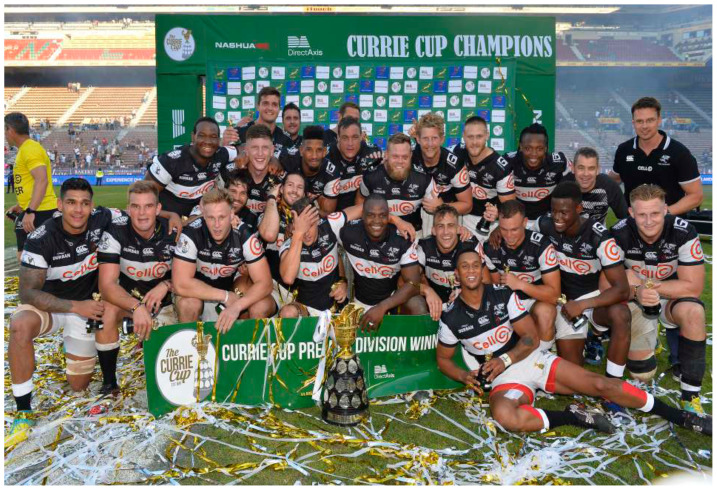


## Executive Summary

As part of the SA Rugby Injury and Illness Surveillance and Prevention Project (SARIISPP), The Currie Cup 2018 Premiership Division Competition (‘The Currie Cup’) injury data were recorded throughout the tournament by the medical doctors of the respective teams. All seven teams were required to record every match injury that occurred in their team. Only match injuries and match time exposure for each participating team were considered for this report.

The Currie Cup injury rate in 2018 was similar to *new* Time-Loss injuries reported in a summary of the international studies[1]. However, the average severity was greater in The Currie Cup, and the overall rate of the *recurrent* injuries was lower than these measurements in the international summary[1].

The injury rate of Time-Loss injuries for The Currie Cup 2018 was 82 (64 to 100) injuries per 1000 player hours (mean and 95% confidence intervals – see p10 for explanation), which is similar to the international rate, 81 (63 to 105) injuries per 1000 player hours[1], and equates to around 1.6 injuries per team per match (i.e. 3 injuries per team every 2 matches).

DHL Western Province had the highest injury rate for Time-Loss injuries for the 2018 tournament and this was significantly higher than the injury rate for the Vodacom Blue Bulls and Cell C Sharks, but not higher than the tournament average. The Cell C Sharks had the lowest injury rate for Time-Loss injuries for the 2018 tournament. DHL Western Province experienced a significant increase in injury rate from 2017 to 2018, while the iCollege Pumas experienced a significant decrease in injury rate from 2017 to 2018.

During The Currie Cup 2018 tournament there were less injuries estimated to be of *minimal* severity, and more estimated to be *moderate* and *severe* in comparison to previous years. After comparing the actual severity of injuries against the estimations, the data revealed that doctors overestimated the *less severe* injuries, and underestimated the *severe* injuries. The average severity of Time-Loss injuries in the 2018 tournament was 31 days, which is lower than the 37 days reported in the England Professional Rugby Injury Surveillance Project, 2018 report[2]. The median injury severity of all Time-Loss injuries was 11 days with 25% of injuries lasting for 5 days or less and 25% of injuries lasting for 35 days or more.

DHL Western Province had the highest number of Time-Loss injuries but were second lowest on *average* severity. The Cell C Sharks had the lowest number of Time-Loss injuries, but were highest on *average* severity. This finding is interesting given that these teams competed against each other in the final and thus equally played the most matches in the tournament.

There were three injuries removed from the severity analysis due to the specific nature of these injuries and the large level of inaccuracy in trying to determine their severity. It must however, be acknowledged, that removing an injury of such a nature has a substantial knock-on effect on the injury severity calculations, and the interpretation of the involved data must be performed with this in mind.

For the first time Central Nervous System injuries were the most common injury type, with sprained ligament and muscle (rupture/strain/tear) injuries decreasing to the second and third most common injury types, respectively. The head remained the most commonly injured body location and concussion the most common injury diagnosis for the third consecutive year. While the tackle remained the most common injury event for the fifth consecutive year, there were significantly less tackle-related injuries in 2018 than the five year combined tournament average and significantly more scrum and open play related injuries in 2018 than the five year combined tournament average.

An interesting finding in this year’s report was that, while that tackle-related injuries came down, the proportion of injuries sustained in the *Scrum* and *Open play* phases in The Currie Cup 2018 were significantly higher than the average proportion of injuries sustained in those phases in the combined 2014 – 2017 tournaments. *‘Acceleration’* accounted for the majority of the injuries sustained in these phases, with almost all of the ‘acceleration’ injuries being muscle strains to the lower limb region. This is important to note as muscle strain injuries are preventable through appropriate conditioning and prehabilitation exercise.[Fig f22-2078-516x-31-v31i1a6532][Fig f23-2078-516x-31-v31i1a6532]

**Figure f22-2078-516x-31-v31i1a6532:**
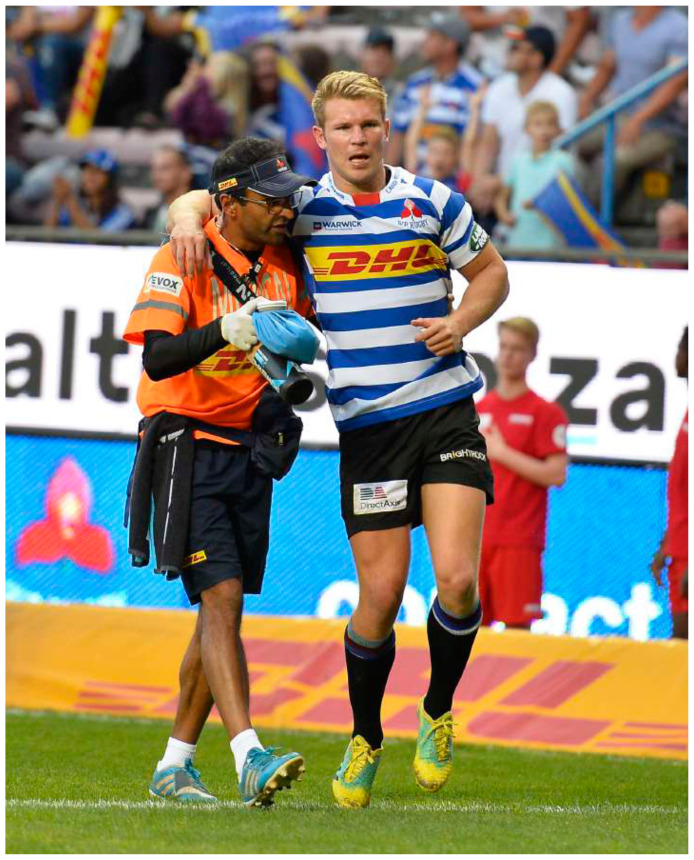


**Figure f23-2078-516x-31-v31i1a6532:**
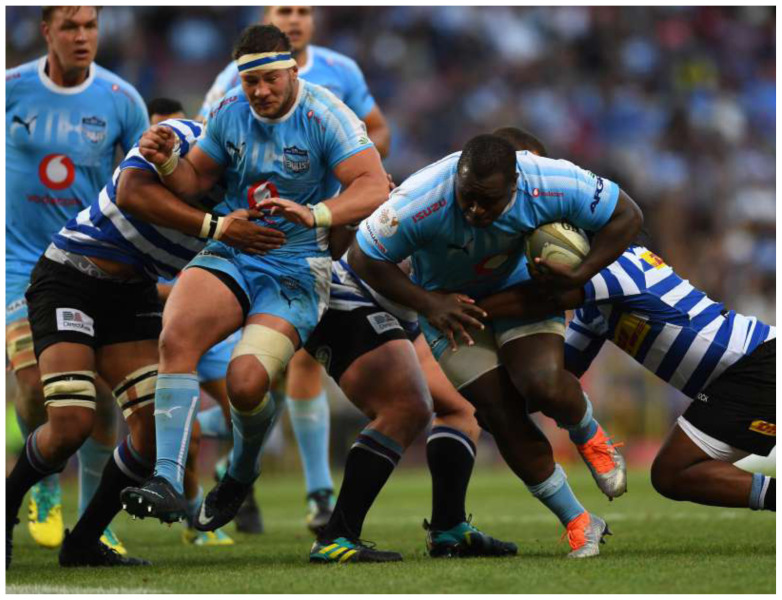


## Introduction

In 2014, as part of the SA Rugby Injury and Illness Surveillance and Prevention Project (SARIISPP), the South African Rugby Union (SA Rugby) implemented a new standardised injury surveillance format for The Currie Cup Premiership Division Competition. This required the team doctor to electronically capture all relevant match injury data on an Application (App) on their cell-phone or tablet. The App provided doctors with the standardised *BokSmart* injury surveillance data capture format, which is based on the international consensus statement [3] for injury recording in rugby union.

Injury surveillance is the critical first step in the development of injury prevention strategies for a particular group. Injury surveillance captured in the correct format enables the comparison of injury rates between teams within the same tournament, tournament injuries over successive years, and with other rugby injury surveillance studies. Literature describing tournament injuries presents the injury numbers as a rate where the total number of injuries is divided by the total amount of time exposed to the risk of experiencing that particular injury. An injury rate is expressed as a number of events per 1000 player hours. This normalised version of the number facilitates comparison between The Currie Cup teams in 2018, previous tournaments and the international injury surveillance literature. Throughout this report the normalised injury rates have been provided to allow for comparison with other tournaments and the international literature, as discussed, but every effort has been made to present these rates on a ‘per team’ and ‘per match’ level for easier and more pragmatic interpretation.

Since 2016, The Currie Cup doctors were asked to record the physical return to play date of the injured players, thereby allowing for the actual severity of the injury to be calculated. For those cases, where the player had not returned to play by the start of the following year, doctors were asked to provide an estimated return to play date. The severity of these particular injuries was then calculated using the estimated date provided, and not the actual date. Calculating the actual severity of most injuries adds substantially to the report as it allows for one to determine the burden of the teams’ injuries with greater accuracy. Injury burden is a combination of the injury rate and severity and is expressed as the number of days’ absent from training and matches per 1000 player hours. There were three injuries removed from the severity calculations for the 2018 report. Two of these injuries were to the iCollege Pumas where the players retired from rugby as a result of the injury they sustained, so no severity could be calculated. The other injury was to the Cell C Sharks where the player suffered a meniscal tear during the Currie Cup 2018, but before recovering from the meniscal tear injury the decision was taken for that player to undergo an ACL reconstruction, which therefore cannot separate the severity of the meniscal injury from time away due to the unrelated surgery. This player was thus recovering from an ACL surgery and not from the meniscal injury he sustained during the competition.

The match played between the Vodacom Blue Bulls and DHL Western Province in round 8 of the competition only consisted of one half due to adverse weather conditions. This shortened match duration has been accounted for in all exposure calculations.

It is important to note that a multitude of factors contribute to players’ injury risk and injury causing events. The medical, conditioning, coaching staff and the players themselves are equally responsible for ensuring that players are medically, mentally and physically fit to handle the demands of the competition. Additionally, each player has unique intrinsic and extrinsic factors that have the potential to increase their risk of injury, which are beyond the team staffs’ control.

An inherent issue with most injury surveillance studies is that the Teams’ Medical doctors are exclusively responsible for entering their team’s injury data. As no audit process is done on the collection of this data in many of these cases , the accuracy of the data is dependent on the compliance of the doctors. This potential limitation is present in most injury surveillance studies. To minimise this potential limitation in this study, SARIISPP had a project coordinator who was in frequent contact with the doctors to ensure they were up-to-date with the data capturing.

In 2018, The Currie Cup semi-finals were between DHL Western Province vs Vodacom Blue Bulls and Cell C Sharks vs. Xerox Golden Lions .The final was between DHL Western Province vs Cell C Sharks, with the Cell C Sharks ultimately winning the tournament.[Fig f24-2078-516x-31-v31i1a6532]

**Figure f24-2078-516x-31-v31i1a6532:**
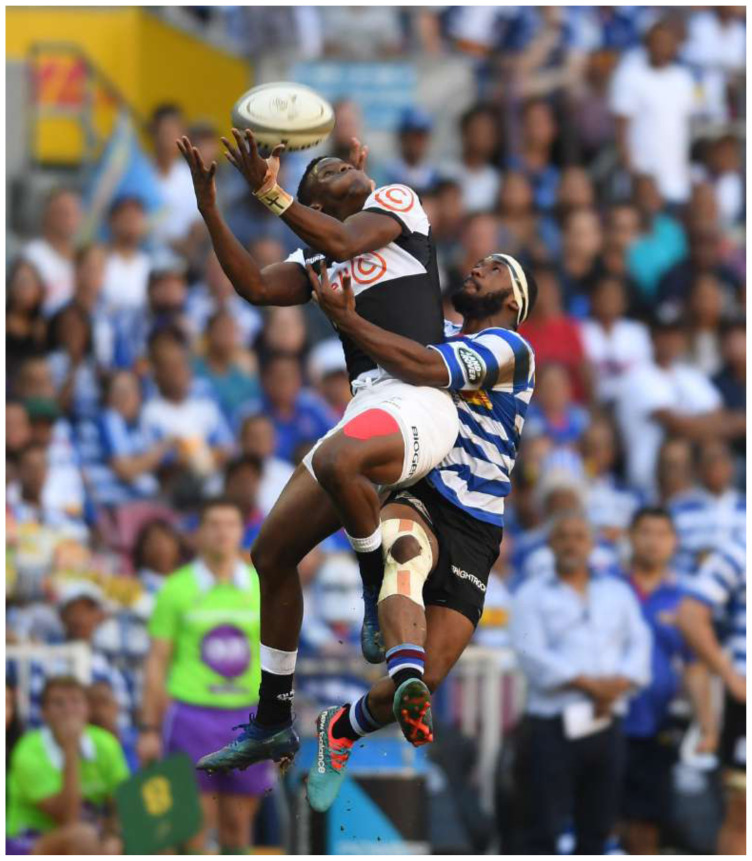


## Definitions

### MEDICAL ATTENTION INJURY

The injury definitions were based on the Consensus Statement of 2007 for injury reporting in rugby union[3]. All injuries that were seen by the Team Medical Doctors were classified as Medical Attention injuries, which are defined by the statement as an “*injury that results in a player receiving medical attention”* [3].

### TIME-LOSS INJURY

Medical Attention injuries were further categorised as Time-Loss injuries, where appropriate, which are defined as “*an injury that results in a player being unable to take a full part in future rugby training or match play*” [3].

### INJURY RATE

For this report, an injury rate is the number of injuries expressed per 1000 player exposure hours. This normalised version of the number of injuries facilitates comparison between The Currie Cup Premiership teams in 2018, previous tournaments and to other international literature. Moreover, the injury rate is expressed as a mean with 95% confidence intervals. A 95% confidence interval around a mean value indicates that there is a 95% chance (i.e. very high chance) that the true value falls within this range. In this report, we are using the approach of examining the overlap of the confidence intervals to determine whether the injury incidences are significantly different; if the range of confidence interval values of two comparisons do not overlap, there is a strong chance (95%) that their injury rates are different from each other. We have opted for this method because it is conservative and less likely to produce false positive results.[4]

### MEDIAN (INTERQUARTILE RANGE)

When values are rank-ordered from lowest to highest, the median is the value which separates the higher half of the values from the lower half of the values. Simply put, it is the middle value of a list of numbers. The interquartile range (IQR) describes the spread of the data. When rank-ordered data is divided into quartiles the first and the third quartile represents the value under which 25% and 75% of the data points fall respectively. As an example, a team may have a median injury severity of 32 days (IQR 7 to 40). This means that when the teams’ injury severities are rank-ordered the mid-point or median of the injury severities is 32 days, with 25% of their injuries resulting in 7 or less days absent from training and matches and 25% of their injuries resulting in 40 days or more absent from training and matches.

### NEW, SUBSEQUENT AND RECURRENT INJURIES

In 2018, in The Currie Cup Premiership Division Competition, a ‘*New Injury’* was defined as when a player sustained his first injury. Any injury that the *same* player sustained after this initial injury was defined as a *‘Subsequent Injury’*.

Subsequent injuries were then further classified into one of four groups based on the OSICS classification diagnosis:

- Different site - Different type
- Different site - Same type
- Same site - Different type
- Same site - Same type

According to the Consensus Statement, any subsequent injury classified as ‘Same site - Same type’ was a *‘Recurrent injury’*.

### INJURY SEVERITY

The total severity of an injury is defined as *“the number of days that have elapsed from the date of injury to the date of the player’s return to full participation in team training and availability for match selection”* [3]. Furthermore severity can be grouped into *Slight* (0–1 days lost), *Minimal* (2–3 days lost), *Mild* (4–7 days lost), *Moderate* (8–28 days lost), *Severe* (>28 days lost), *Career ending* and *Non-fatal catastrophic* [3].

The average severity represents the average number of days lost per injury when dividing the accumulated total number of days lost by the total number of injury events. For example, a team may have a total severity of 550 days absent, accumulated from 22 injuries. The average severity of the team’s injuries would therefore be 550/22, which equals, on average 25 days absent per injury.

### INJURY BURDEN

Injury burden is a combination of injury rate and severity. It is the injury rate multiplied by the average severity (number of days lost due to injury) and is expressed as the number of days absent per 1000 player hours. For example, a team who has an injury rate of 75 injuries per 1000 player exposure hours, and an average severity of 38 days lost per injury will have an injury burden of 2850 days absent per 1000 player hours (75 x 38).

### OPERATIONAL INJURY BURDEN

The operational burden is the expected number of days lost per injury per team for every match played over the tournament or season. The measure is an extrapolation of injury rates and severities over a season, and includes the most severe injuries together with the least severe injuries in its estimation. For example, if a team has an operational injury burden of 2 days, it means that based on their injury rates and average severity, on average, 2 days absence can be expected from every match injury the team sustains.

### META-ANALYSIS

A meta-analysis is a study using statistical methods to combine multiple scientific studies with varying levels of evidence on the same topic to determine overall defining patterns and results from the combined data. As such, it represents the highest level of scientific evidence available. The findings in this report are compared to that of the most recent meta-analysis for rugby union injuries at a senior professional level [1].

## Key Findings

### Injured players

Over the course of The Currie Cup 2018, 66 players sustained a total of 77 Time-Loss injuries. This means that of the 154 players exposed to playing rugby in the tournament (7 teams x 22 player match-day squad), 43% sustained an injury at some point (Figure 1a). The proportion of players who experienced one Time-Loss injury has increased from 2017 to 2018, while the proportion of players who experienced more than one injury has decreased from 2017 to 2018 (Figure 1b). Further analyses will focus on absolute injury numbers, regardless of the number of players who sustained them.

### Overall injury rate

Only the number of Time-Loss injuries, those which resulted in the player missing more than one training session/match, will be considered for analyses, based on the fact that these injuries are more comparable between different teams, tournaments and international literature [3].

Overall, the match injury rate for all Time-Loss injuries in 2018 for The Currie Cup was 82 (64 to 100) injuries per 1000 player exposure hours. This is almost identical to the rate of the meta-analysis (81 injuries per 1000 player hours, 63 to 105) [1]. An injury rate of 82 injuries per 1000 player hours equates to 1.6 injuries per team per match or 3 injuries every two matches.

When ranking the teams from highest to lowest injury rate for Time-Loss injuries, the DHL Western Province had the highest injury rate and the Cell C Sharks had the lowest injury rate. These teams both competed in the final and thus both played the most matches of all teams. The Time-Loss injury rate of the DHL Western Province is significantly higher than that of the Vodacom Blue Bulls and Cell C Sharks, but not significantly higher than the tournament average (Figure 2a).

The average severity of match injuries for The Currie Cup 2018 was 31 days, which is slightly lower than the average severity for match injuries of 37 days reported in the England Professional Rugby Injury Surveillance Project, 2017 – 2018 season report [2]. The median severity of all Time-Loss injuries was 11 days (IQR 5 to 35). This means that the half-way mark of the injury severities was 11 days, with 25% of all Time-Loss injuries lasting for 5 days or less and 25% lasting for longer than 35 days.

Table 1 displays the injury rates of Time-Loss injuries of each team (depicted in Figure 2a), as a number of incidents per match and per player. The Vodacom Blue Bulls have been used as a worked example to explain the table. The Vodacom Blue Bulls had an injury rate of 46 injuries per 1000 player hours, which equates to 1.1 injuries per match. Another way to describe this is, for every 21.7 player match hours the Vodacom Blue Bulls sustained one match injury. At the start of each match if all players had an equal chance of injury, each player in the Vodacom Blue Bulls team would have a 6% chance of injury for each match played. With a 6% chance of injury at each match, a player in the Vodacom Blue Bulls team can expect to be injured once in every 16.3 matches played (Table 1). The information in this table will be unfolded in the subsequent graphs.

When comparing the teams Time-Loss injuries for The Currie Cup 2014 – 2018 tournaments (Figure 2b), DHL Western Province experienced a significant decrease in injury rate in 2017 followed by a significant increase in 2018, while the iCollege Pumas experienced a significant increase in injury rate in 2017 and a subsequent significant decrease in 2018.

It is interesting to note that the combined mean and 95% CI for all teams for all years, 82 (75 to 88) injuries per 1000 player hours is similar to the summary of international data described in the meta-analysis[1], 81 (63 to 105) injuries per 1000 player hours.[Fig f25-2078-516x-31-v31i1a6532]

**Figure f25-2078-516x-31-v31i1a6532:**
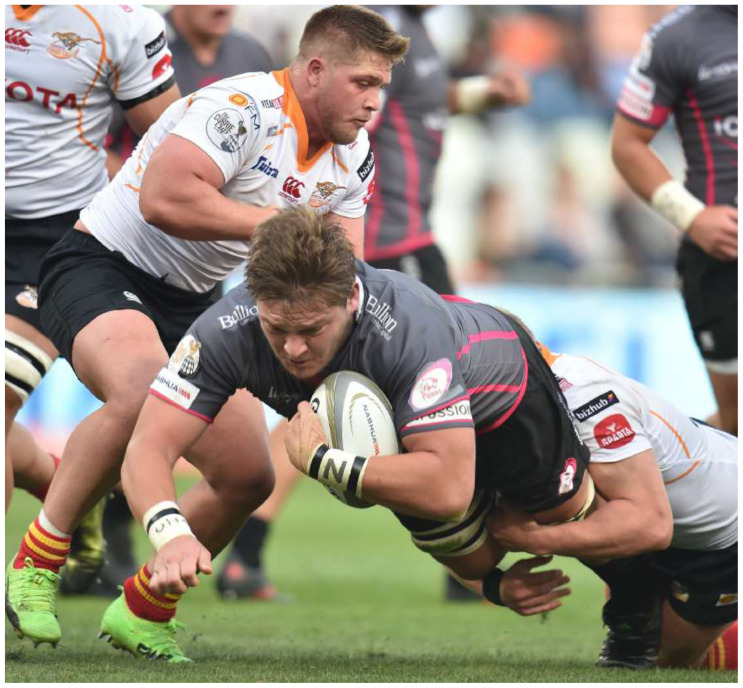


### Average Time-Loss Injury rates per Month of the Tournament

**Figure 3:**
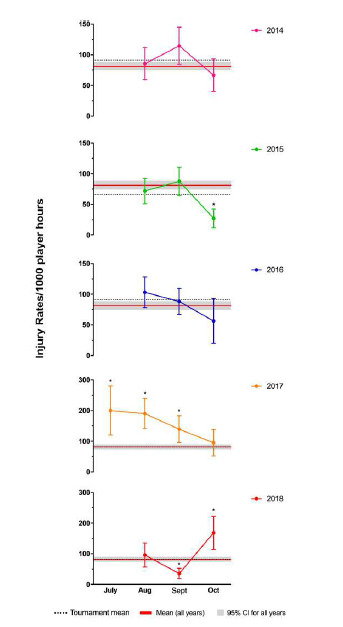
Average Time–Loss Injury rate per month for The Currie Cup 2014 – 2018 tournaments with mean and 95% confidence intervals (CI) for all teams for all years combined. Asterisk (*) indicates injury rates are significantly different to the mean tournament average.

In 2018 the opposite trend was observed to previous years, with the injury rate being the lowest in the middle of the tournament and highest in the final month. The injury rate in September was significantly lower, and the injury rate in October was significantly higher, than the tournament average of all years combined (Figure 3).

### New, Subsequent and Recurrent Injuries

Overall, the injury rate for New Time-Loss injuries for The Currie Cup 2018 was 70 (53 to 87) injuries per 1000 player hours, which is similar to that of the meta-analysis[1] with a rate of 78 (74 to 83) injuries per 1000 player hours. The average severity for New Injuries for The Currie Cup 2018 was 34 (23 to 46) days, which is higher than the average severity reported in the meta-analysis[1] of 20 (15 to 24) days.

There were 8 players who sustained more than one injury in The Currie Cup 2018. The majority (73%) of the subsequent Time-Loss injuries were at a different anatomical site and of a different type to the first injury (Figure 4a & 4b).

A recurrent injury was any subsequent injury classified as ‘same site-same type’. There were only two recurrent injuries in The Currie Cup 2018. The overall injury rate for recurrent Time-Loss injuries was 2 (1 to 5) injuries per 1000 player hours, which is significantly lower than the meta-analysis rate of 11 (10 to 12) injuries per 1000 player hours[1]. The severity of the two recurrent injuries in 2018 was 3 and 9 days lost respectively.

**Figure 5:**
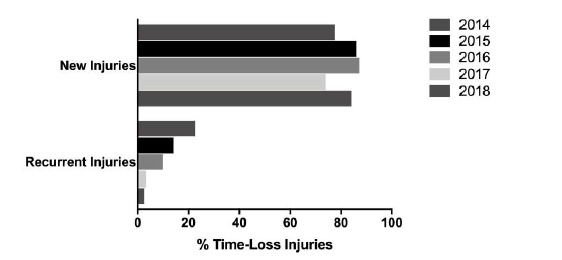
Proportion of new versus recurrent injuries for The Currie Cup 2014 – 2018 tournaments. Data expressed as a % of Time-Loss injuries for 2014 (n=120), 2015 (n=114), 2016 (n=142), 2017 (n=126) and 2018 (n=77).

When comparing the new and recurrent injuries across The Currie Cup 2014 – 2018 tournaments, there was a decrease in the proportion of recurrent injuries over the five years (Figure 5). This finding must be interpreted with caution as in 2014 and 2015 the classification of a recurrent injury was made by the doctor, thus the definition of a recurrent injury was open to interpretation by the doctor. In 2016, 2017 and 2018 however, an injury was classified as a recurrent injury based on the OSICS classification diagnosis [5].[Fig f26-2078-516x-31-v31i1a6532]

**Figure f26-2078-516x-31-v31i1a6532:**
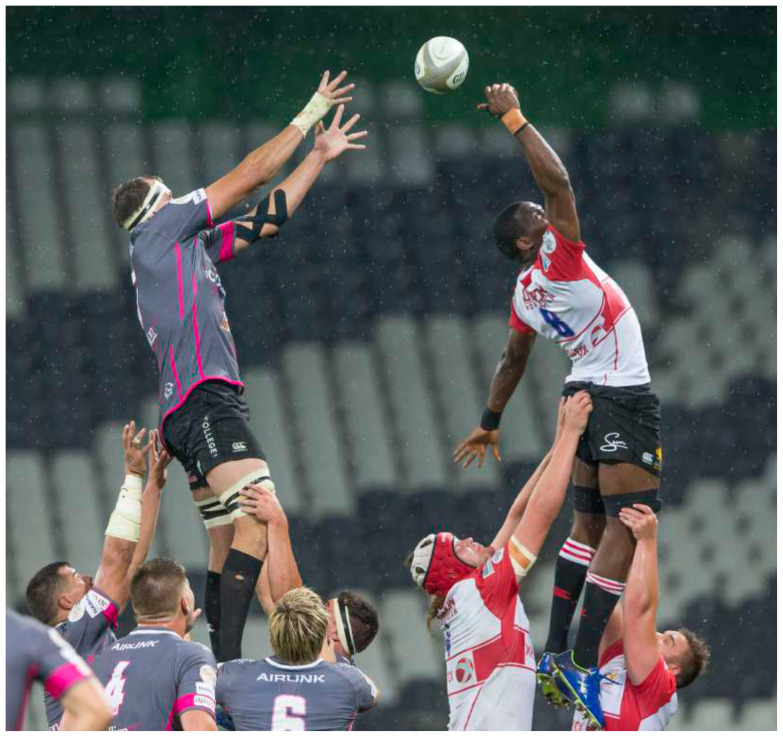


### Injury Severity

For each year, at the time of injury the Team Medical doctors were asked to estimate the severity of the injury based on their clinical assessment of the injured player. A *‘Minimal’* injury refers to 2–3 days (and includes *‘Slight’* injuries of 0–1 days – see subsequent paragraph), *‘Mild’* is 4–7 days, *‘Moderate’* is 8–28 days and *‘Severe’* is >28 days off rugby training and/or match play.

The doctors estimated some Time-Loss injuries as having *Slight* severity. However, according to the consensus statement definition most *Slight* injuries are by definition ‘Medical Attention’. Therefore, the *Slight* injuries that met the Time-loss definition, were recorded as ‘Time-Loss’ by the team doctors and were then added into the higher severity category of *Minimal* for the purpose of this analysis [3].

**Figure 6:**
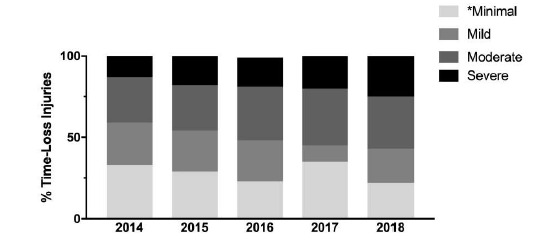
The estimated severity of Time-Loss injuries from all participating teams for The Currie Cup 2014 (n=120), 2015 (n=114), 2016 (n=141), 2017 (n=126) and 2018 (n=77) Competitions. * ’Minimal’ includes Time-Loss injuries which were reported as ‘Slight’ or ‘Minimal’.

Figure 6 compares the estimated injury severity of The Currie Cup 2014 – 2018 tournaments. There were no significant differences in the estimated injury severity between the five years (Figure 6).

**Figure 7:**
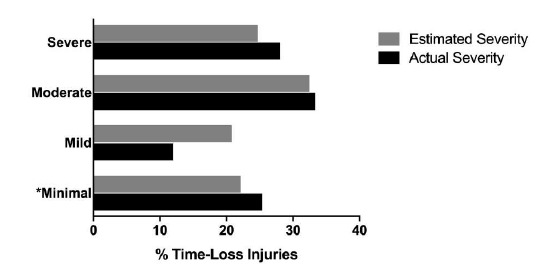
The estimated and actual severity of Time-Loss injuries for The Currie Cup 2018. Data expressed as a proportion of all Time-Loss Injuries (Estimated n=77; Actual n=74). * ’Minimal’ includes Time-Loss injuries which were reported as ‘Slight’ and ‘Minimal’.

Since The Currie Cup 2016, doctors were asked to record the physical return to play date of the player following the injury, which allowed for the actual injury severity to be calculated. Figure 7 compares the estimated severity recorded by the doctor at the time of injury and the actual injury severity determined once the injured player had returned from injury. In The Currie Cup 2018, doctors over-estimated the number of *‘Mild’* injuries and under-estimated the number of *‘Severe’* injuries. Doctors estimated 25% of Time-Loss injuries to be *‘Severe’* while 29% of Time-Loss injuries were *‘Severe’*.

Table 2 describes the actual severity of each teams’ Time-Loss injuries for The Currie Cup 2018. The Vodacom Blue Bulls have again been used as a worked example to explain the table. The Vodacom Blue Bulls sustained 0.9 injuries per match, meaning that for every 1.1 matches played they sustained one injury. In total, the Vodacom Blue Bulls lost 143 training and match days due to injury. This equates to an average of 24 training and match days lost for every injury sustained. The burden of the team’s injuries equates to 1096 days lost per 1000 player hours. Translating this to an operational burden per match, it shows that the Vodacom Blue Bulls lost 21 days per injury per match over the season. The median injury severity for the Vodacom Blue Bulls was 24 days (IQR 11 to 36). This means that when severities of the Vodacom Blue Bulls Time-Loss injuries were rank-ordered the midpoint of the severities was 24 days off from rugby, with 25% of their injuries lasting equal to or less than 11 days off and 25% of their injuries lasting equal to or longer than 36 days off (Table 2).

**Figure 8:**
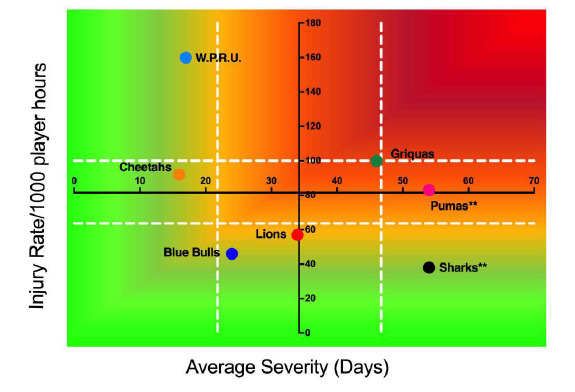
Injury rate plotted against the average severity of Time-Loss injuries for each participating team in The Currie Cup 2018. The Y-axis Average Injury Rate is expressed as the tournament average (±95% CI) and X-axis Average Severity is expressed as the average (±95% CI) of the individual injury severities in the tournament.**Injuries removed from severity calculations. Pumas n = 2, Sharks n = 1

DHL Western Province had the highest rate of Time-Loss injuries, but these were of low severity. Conversely the Cell C Sharks had a low injury rate, but their injuries were of high severity (Figure 8). Teams who fall in the green zone, will generally not be impacted as much by their injury burden, regardless of whether their injury rate or average severity is relatively high. As soon as the combination of rate and severity moves into the orange and/or red zone, the impact on team performance and player availability becomes more problematic.

### Injury Type

Central Nervous System injuries (25%, n=19) were the most common Time-Loss injuries recorded in The Currie Cup 2018, with sprained ligament injuries comprising the second highest proportion (23%, n=14). The Central Nervous System injuries were comprised of 14 concussions and 5 nerve injuries. Of the 5 nerve injuries, 4 were to the shoulder and 1 to the cervical spine. Central Nervous System injuries had an injury burden of 427 days absent/1000 player hours, which equates to an operational injury burden of 9 days lost per Central Nervous System injury per match over the season. The median severity for Central Nervous System injuries was 12 days with 25% of Central Nervous System injuries resulting in 9 or less days absent from training and matches, and 25% of injuries resulting in 16 or more days absent from training and matches (Table 3).

**Figure 9:**
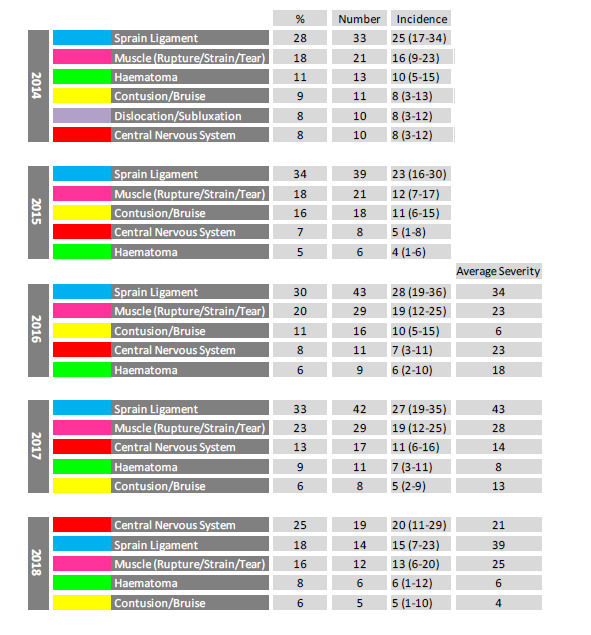
The movement of the most common types of Time-Loss Injuries from 2014 – 2018. Data expressed as a %, absolute number and incidence of Time-Loss injuries for 2014 (n=120), 2015 (n=114), 2016 (n=142), 2017 (n=126), 2018 (n=77) and the average severity for 2016, 2017 and 2018 is expressed in days.

When looking at the most common injury types over the five years, this is the first year that sprained ligaments and muscle injuries have not been the first and second most common injury types. Both the absolute number and proportion of Central Nervous System injuries has increased gradually since 2015, with Central Nervous System injuries being the most common injury type in 2018 (Figure 9).

**Figure 10:**
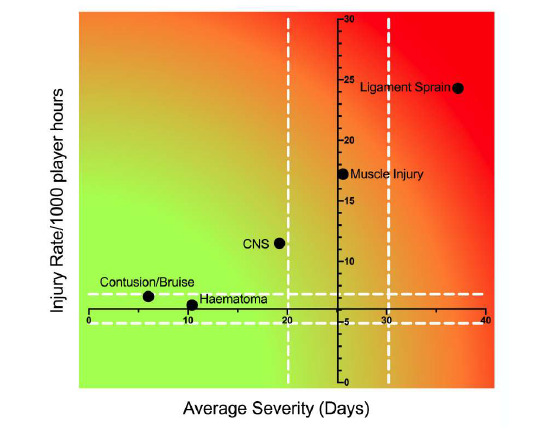
Injury rate plotted against the average severity of the most common Time-Loss injury types combined for 2016 to 2018. The Y-axis Average Injury Rate is expressed as the combined average for the plotted injuries (±95% CI) and X-axis Average Severity is expressed as the average of the individual injury severities for the plotted injuries (±95% CI).

Combining the five most common injury types from 2016 – 2018 revealed that ligament sprain injuries are both the most frequently occurring injury type and have the highest average severity. While not as high as ligament injuries, muscle injuries also have a high injury rate and average severity (Figure 10). The effective burden of these two injury types still dominate, and therefore impact teams more than the other injury types do.

For further comparison, The Currie Cup 2018 Time-Loss injury types were grouped in a similar way to the meta-analysis of the international studies[1]. With these groupings, aligned to the meta-analysis, the most common Time-Loss injury types in The Currie Cup 2018 were joint (non-bone)/ligament injuries.

The injury rate of 20 (11 to 29) injuries per 1000 player hours for the central nervous system during The Currie Cup 2018, was higher, although not significantly so, than the rate of “central/peripheral” system injuries of the meta-analysis, 8 (4 to 15) injuries per 1000 player hours[1].

The injury rate for muscle/tendon injuries (comprised of muscle rupture/strain/tear, tendon injury/rupture and tendinopathy) was 17 (9 to 25) injuries per 1000 player hours. This was lower than the same type of injury grouping in the meta-analysis, which had a rate of 40 (21 to 76) injuries per 1000 player hours, albeit not significant[1]. The average severity for muscle/tendon injuries of 38 (8 to 69) days, in The Currie Cup 2018 was higher than the average severity of 15 (5 to 24) days reported in the meta-analysis[1], but this was not significantly different.

In contrast, joint (non-bone)/ligament injuries (comprised of dislocation/subluxation and sprain/ligament) were comparable: 25 (15 to 35) injuries per 1000 player hours in the present study, compared to the 24 (18 to 65) injuries per 1000 player hours in the meta-analysis[1]. The average severity of joint (non-bone)/ligament injuries in The Currie Cup 2018 was 53 (29 to 78) days, which is again higher, but not significantly different, to the average severity for the same types of injuries reported at 29 (19 to 39) days in the meta-analysis[1].

It appears that while the injury rates of these grouped injury types for The Currie Cup 2018 were similar to the injury rates reported in the meta-analysis, the average severity of The Currie Cup injuries was higher, although again, this was not significantly different.[Fig f27-2078-516x-31-v31i1a6532]

**Figure f27-2078-516x-31-v31i1a6532:**
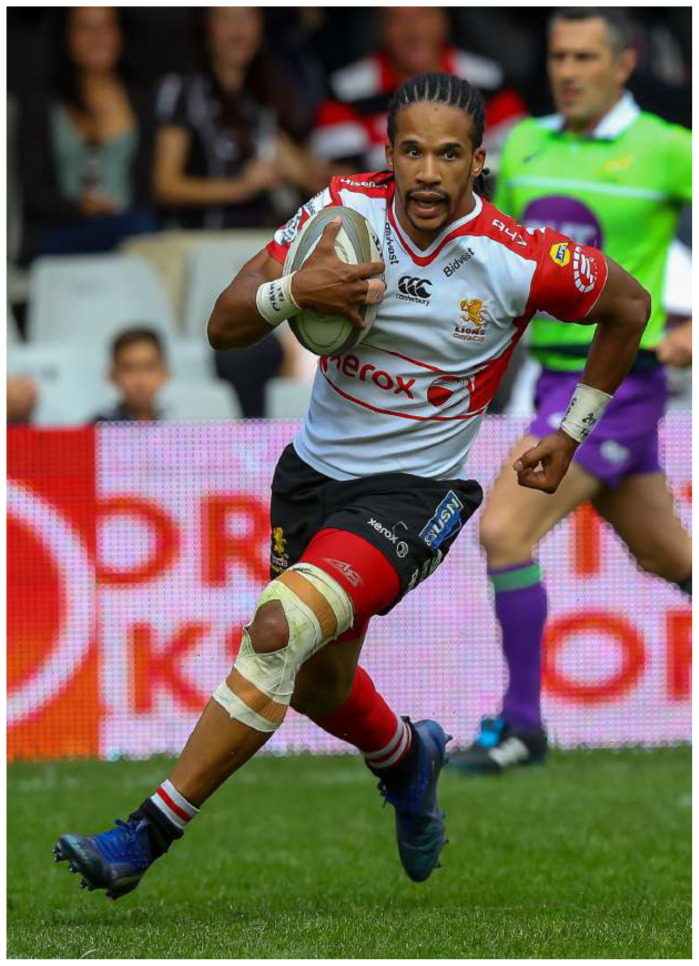


### Injury Diagnosis[5]

**Figure 11:**
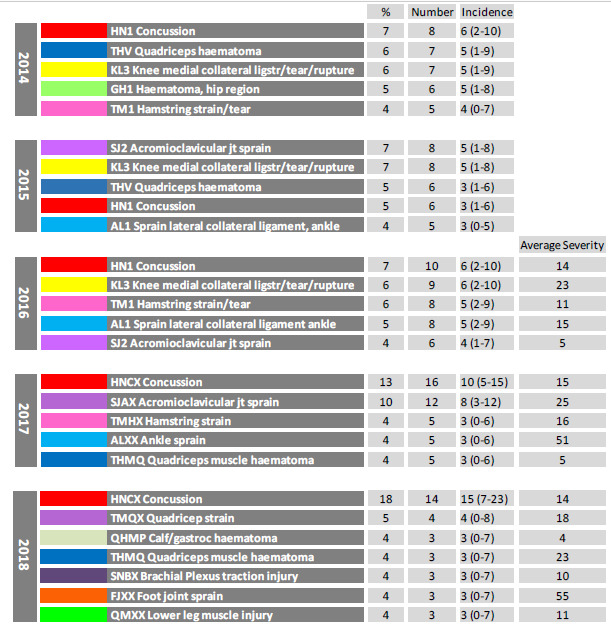
The movement of the most common OSICS classification diagnoses [5] of Time-Loss Injuries from 2014 – 2018. Data expressed as a %, absolute number and incidence of total Time-Loss injuries for 2014 (n=120), 2015 (n=114), 2016 (n=142), 2017 (n=126) and 2018 (n=77); the average severity for 2016, 2017 and 2018 is expressed in days.

The most frequent OSICS classification diagnosis^5^ in The Currie Cup 2018 was HNCX Concussion. Concussions were also the most frequently diagnosed injury in the 2014, 2016 and 2017 tournaments (Figure 11).

### Concussions

**Figure 12:**
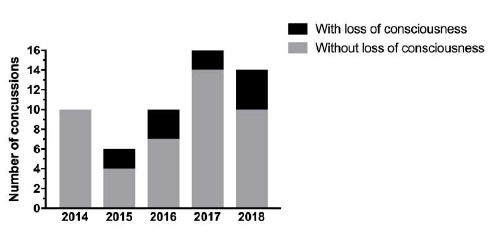
Number of concussions with and without loss of consciousness for The Currie Cup 2014 (n=10), 2015 (n=6), 2016 (n=10), 2017 (n=16) and 2018 (n=14) Premiership tournaments.

Concussions made up 18% (n=14) of all Time-Loss injuries for The Currie Cup 2018. Of these 14 concussions, 4 were recorded with a loss of consciousness (Figure 12). The average severity of concussions reported in the 2018 tournament was 14 days (Range 4 – 38 days), which is slightly lower than the average severity of 19 days reported for concussion in the England Professional Rugby Injury Surveillance Project, 2017 – 2018 season report[2].[Fig f28-2078-516x-31-v31i1a6532]

**Figure f28-2078-516x-31-v31i1a6532:**
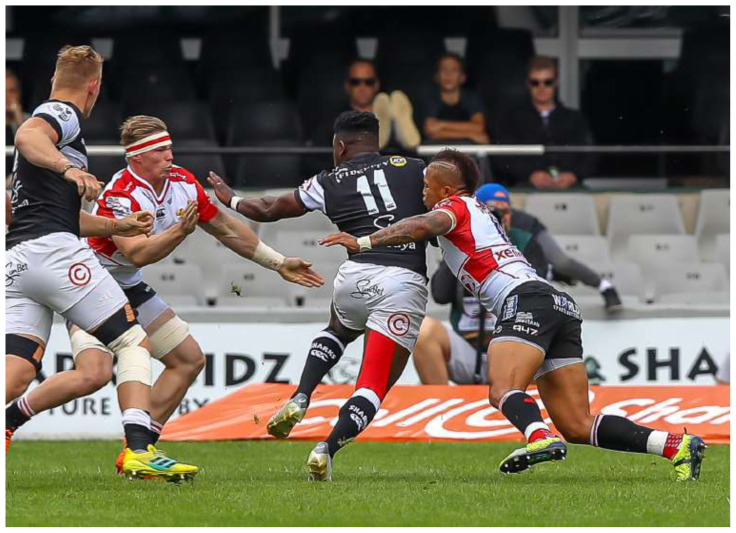


### Region of Injury

The most frequently injured body location of all Time-Loss injuries during The Currie Cup 2018 was the Head (18%, n=14), followed by the Shoulder (10%, n=8) and Knee (10%, n=8). The average burden of head injuries in 2018 was 206 days absent per 1000 player hours. This translates to an operational burden of 4 days lost per head injury per match over the entire season. The median severity of head injuries in 2018 was 12 days absent, with 25% of head injuries resulting in 9 or less days lost from training and matches and 25% of all head injuries resulting in 16 or more days lost from training and matches (Table 4).

**Figure 13:**
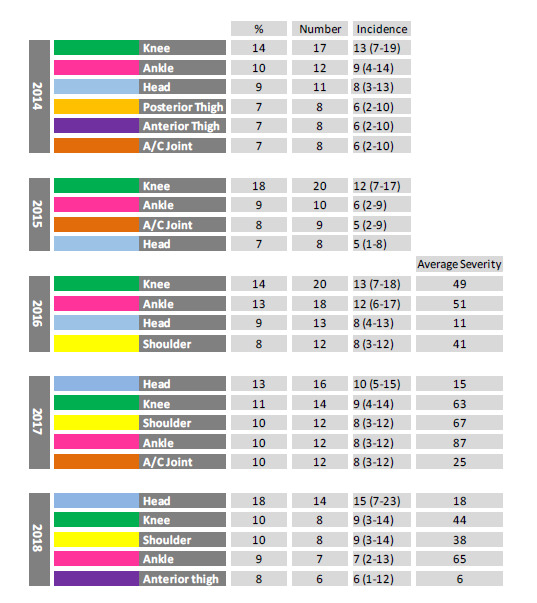
The movement of the most common body locations of Time-Loss Injuries from 2014 – 2018. Data expressed as a %, absolute number and incidence of total Time-Loss injuries for 2014 (n=120), 2015 (n=114), 2016 (n=142), 2017 (n=126), 2018 (n=77) and the average severity for 2016 – 2018 expressed in days.

When looking at the movement of the most common body locations of Time-Loss injuries for The Currie Cup 2014 – 2018 tournaments it is interesting to note that the top four most common injury locations were the same in 2017 and 2018. The head remained the most commonly injured body location in 2018, and even though the absolute number dropped by 2, the proportion and average severity of injuries to the head increased in 2018 from 2017 (Figure 13).

In the Currie Cup 2017 report the gradual increase in proportion of injuries to the shoulder was highlighted as a potential area of concern given the high average severity these injuries carry. It is pleasing to note that while the shoulder remains the second most commonly injured body location in the 2018 tournament, the proportion of these injuries has decreased by 1% and the average severity (38 days lost) is the lowest that has been recorded across the 2016, 2017 and 2018 tournaments.

**Figure 14:**
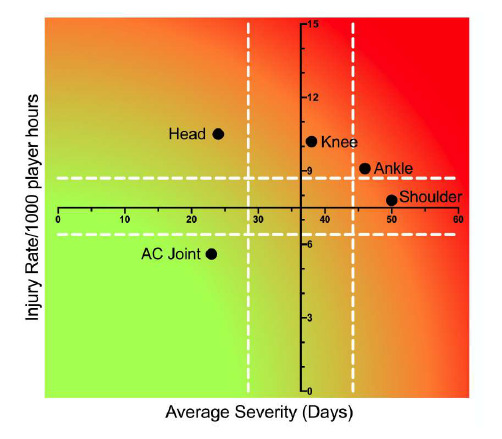
Injury rate plotted against the average severity of the most common body locations of Time-Loss injuries combined for 2016 to 2018. The Y-axis Average Injury Rate is expressed as the combined average for the plotted injuries (±95% CI) and X-axis Average Severity is expressed as the average of the individual injury severities for the plotted injuries (±95% CI).

Combining the most commonly injured body locations from 2016 – 2018 revealed that injuries to the shoulder have a moderate injury rate, but are of a high average severity. Conversely, injuries to the head have a high injury rate, but do not carry a high average severity (Figure 14). Knee and ankle injuries due to their higher burden of injury, also require attention.

**Figure 15:**
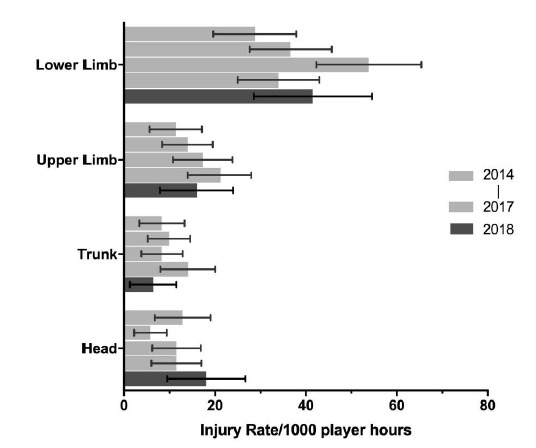
The Time-Loss injury rates of the four most common grouped body locations (injuries/1000 player hours) for 2014 (n=114), 2015 (n=120), 2016 (n=142), 2017 (n=126) and 2018 (n=77).

When anatomical body locations were grouped for comparison with the data from the meta-analysis[1], the lower limb had the highest injury rate for The Currie Cup 2018. In 2018, the injury rate to the lower limb of 42 (9 to 55) injuries per 1000 player hours was similar to the meta-analysis[1] at 47 (28 to 46) injuries per 1000 player hours. The injury rate to the lower limb has increased from 2017 to 2018. The lower limb also had the highest average severity of 47 days, followed by the upper limb at 30 days lost. (Figure 15).

### Injury Event

In The Currie Cup 2018, *tackling* (i.e. performing the tackle) accounted for the highest proportion (29%, n=22) of injury events. *Tackling* in the 2018 tournament had an injury rate of 23 (14 to 33) injuries per 1000 player hours. This is comparable to the meta-analysis[1] injury rates of 19 (12 to 29) injuries per 1000 player hours. *Being tackled* (i.e. to the ball carrier) had an injury rate of 12 (5 to 19) injuries per 1000 player hours, which is lower than the injury rate for ball carriers in the meta-analysis of 29 (19 to 46) injuries per 1000 player hours. The trend in 2018 where *tackling* (i.e. performing the tackle) accounted for more injuries than *being tackled* (i.e. to the ball carrier) is opposite to the trend seen in the meta-analysis[1].

In The Currie Cup 2014 – 2018 tournaments, the tackle phase accounted for the highest proportion of all injury events (Figure 16a). While the tackle-related injuries came down, the proportion of injuries sustained in the *Scrum* and *Open play* phases in The Currie Cup 2018 were significantly higher than the average proportion of injuries sustained in those phases in the combined 2014 – 2017 tournaments (Figure 16a). *‘Acceleration’* accounted for 60% (n=9) of injuries in the Scrum and 63% (n=12) of injuries in Open play in The Currie Cup 2018. Expanding on the *‘acceleration’* injuries reveals that in the *Scrum*, 7 of 9 injuries were to the lower limb, of which 5 were muscle strains. In *Open* play, 10 of the 12 injuries were to the lower limb, of which 5 were muscle strains. This is important to note as muscle strain injuries are preventable through appropriate conditioning and prehabilitation exercise.[Fig f29-2078-516x-31-v31i1a6532][Fig f30-2078-516x-31-v31i1a6532]

**Figure f29-2078-516x-31-v31i1a6532:**
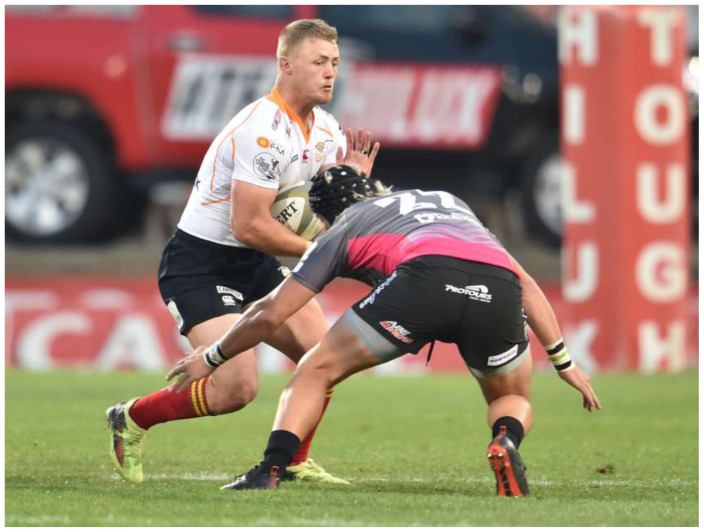


**Figure f30-2078-516x-31-v31i1a6532:**
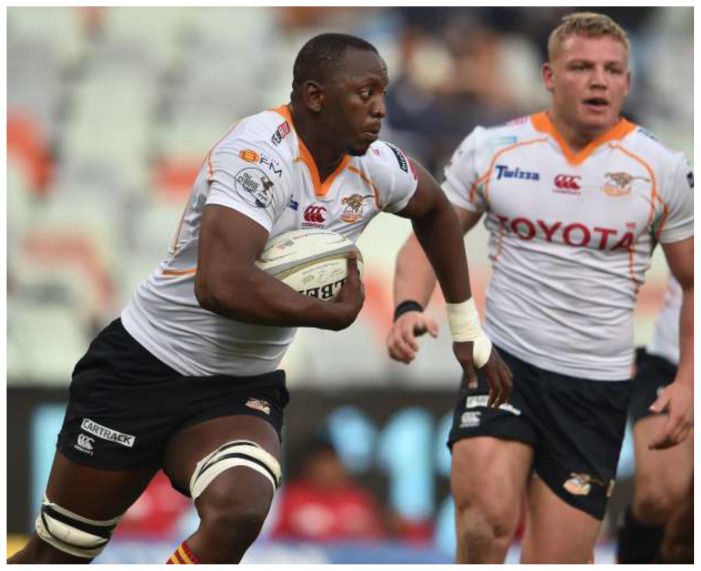


When assessing the mechanism of tackle-related injuries, tackling front-on accounted for the highest proportion of tackling related injuries in 2014 (44%, n=23), 2015 (33%, n=13), 2016 (30%, n=17), 2017 (30%, n=13) and 2018 (35%, n=8) (Figure 16b).

### Playing Position of Injured Players

In The Currie Cup 2018, forwards had a Time-Loss injury rate of 96 (67 to 125) injuries per 1000 player hours, while for the backs it was 66 (44 to 89) injuries per 1000 player hours. When comparing these rates with the meta-analysis[1], the 2018 rate for forwards was comparable with the meta-analysis, while the rate for the backs was lower than the 99 (92 to 106) injuries per 1000 player hours reported in the meta-analysis.

When divided into specific positional groupings within forwards and backs, the number of injuries were normalised relative to the number of players on the field per position per team (e.g. 2 Props = total injuries divided by 2; 3 Loose Forwards = total injuries divided by 3). However, with the relatively low numbers, it is difficult to draw any firm conclusions.

### Protective Gear and Injury

**Figure 18:**
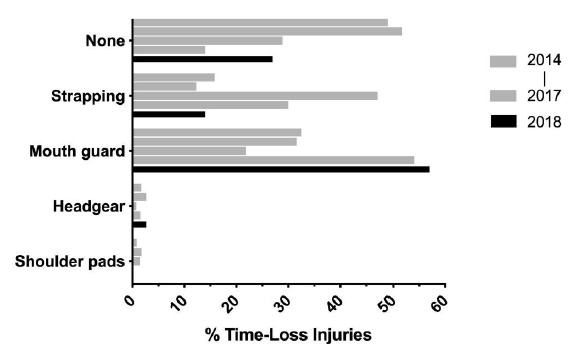
The protective gear worn by players when sustaining a Time-Loss injury for The Currie Cup 2014 (n=120), 2015 (n=114), 2016 (n=142), 2017 (n=126) and 2018 (n=77) tournaments.

The majority (57%) of all players who sustained a Time-Loss injury in The Currie Cup 2018 were wearing a mouth guard. Of the 14 concussions, four of them occurred with the player wearing a mouth guard. The number of players who participated in the match wearing a mouth guard and those who did not is unknown. Therefore, one cannot draw any firm conclusions. In 2014 and 2015 most of the injured players were not wearing any form of protective gear (2014 = 49%, 2015 = 52%), while in 2016 the majority of the injured players were wearing strapping (47%) and in 2017 the majority were wearing mouth guards (54%) [Fig f17-2078-516x-31-v31i1a6532](Figure 18).

### Venue

**Figure 19:**
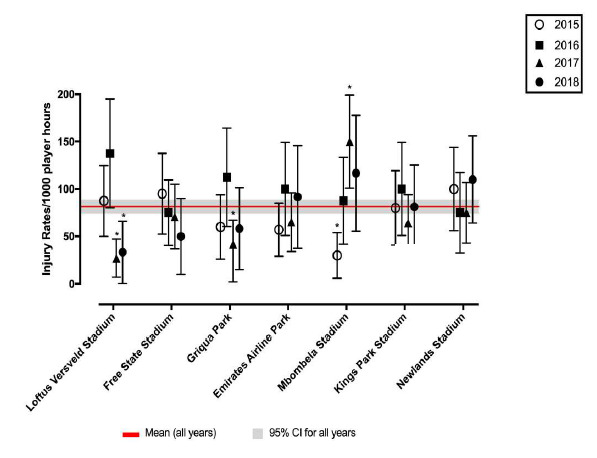
The injury rate of Time-Loss injuries at the seven utilised stadia in 2015 (n=120), 2016 (n=142), 2017 (n=126) and 2018 (n=77). Mean and 95% confidence interval for all injuries combined for 2015, 2016, 2017 and 2018. Asterisks (*) indicates significantly different to combined tournament average.

Matches were played at seven stadia during the tournament. For The Currie Cup 2018, Loftus Versfeld Stadium, Pretoria had the lowest injury rate, which was significantly lower than the combined average injury rate. Mbombela Stadium, Nelspruit had the highest injury rate, but this was not significantly higher than any other stadium or the tournament average (Figure 19).

**Figure 20:**
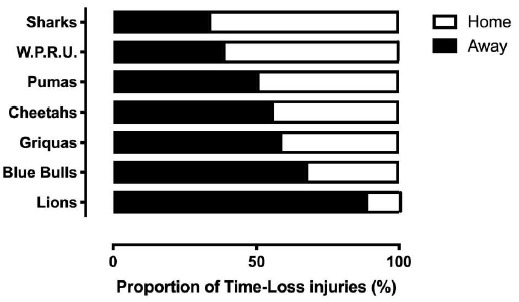
Proportion of injuries sustained playing at home and away venues for The Currie Cup 2018 Premiership Division Competition (n=77).

Across all teams, and although not significant, playing at home (39 [27 to 52] injuries per 1000 player hours) had a lower injury rate than playing away (43 [29 to 56] injuries per 1000 player hours) in The Currie Cup 2018 tournament. The iCollege Pumas sustained an equal number of injuries playing away and at home. The Cell C Sharks and DHL Western Province sustained more injuries playing at home than away, and all other teams sustained more injuries when playing away, especially the Lions and Blue Bulls (Figure 20).[Fig f31-2078-516x-31-v31i1a6532]

**Figure f31-2078-516x-31-v31i1a6532:**
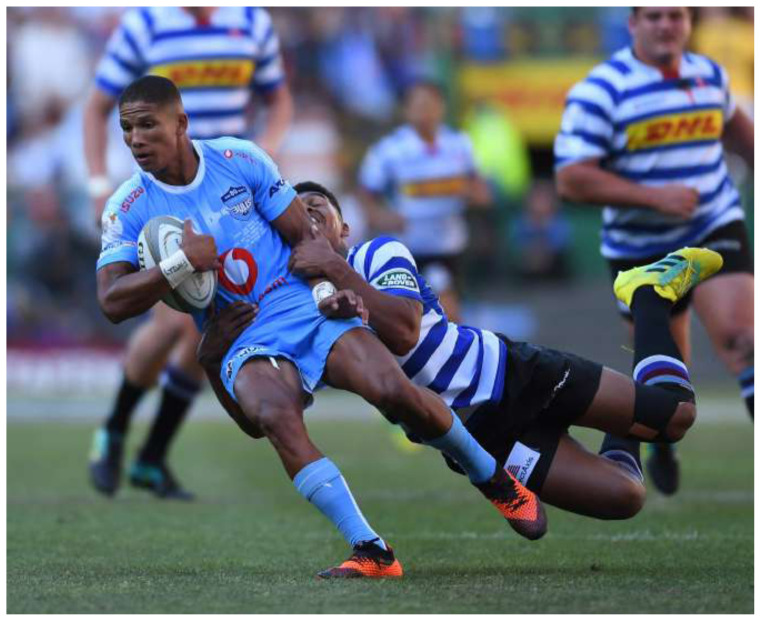


## Thoughts for future research

It is well accepted in the literature that match injury rates are expressed as a rate per 1000 player exposure hours, with match exposure being included as 80 minutes. In a rugby union match however, the ball is only in play for around 40% of this 80 minutes. In the Currie Cup 2018, the average ball in play time was 32 minutes (range 28 – 42 mins). This means that on average players were only exposed to a risk of injury for 32 of the 80 minutes in the match. When the ball is not in play, players are at no risk of injury and use this time to recover before the ball is back in play. It follows that in matches where ball in play time is high, players are at a higher risk of injury. This is both because the player has a greater exposure to match play and because they have less time to recover during the match. As a result they could be playing in a more fatigued state. Presenting injury rates per ball-in-play time would provide a more accurate description of the risk of injury in a match.

Table 5 presents the injury numbers in respect to ball-in-play time for each year of The Currie Cup competition. Only ball-in-play time of teams who have complete injury data has been presented to allow for fair comparison with the traditionally calculated injury rates presented throughout this report. When looking at ball in play time for The Currie Cup competition from 2014 – 2018, there has been no significant change in average ball in play time per match across the years. As such the relative injury incidence trends, when presented as a rate per 1000 ball-in-play time hours, is comparable to the injury incidence when presented as a rate per 1000 match hours. Changes in the competition format across the years to include different numbers of teams and matches played has resulted in a substantial change in average ball in play time per team across the years. As such, teams were exposed to almost double the total ball in play time in 2017 than in 2018. This extra exposure time has potential consequences on player injury risk. Arguably, the most interesting finding from presenting the injury data in respect to ball in play time is the average number of injuries per team per ball in play time minute. If the ball was to be in play for the total 80 minutes of a match, we could expect an average of 4 Time-loss injuries per team per match. This equates to an average of 8 Time-loss injuries per match and means that for any given match one could expect 27% of the fielded players to sustain an injury. This highlights the importance of monitoring the ball-in-play time for the Currie Cup competition as any significant change in ball in play time contributes directly to the players’ risk of injury.

## Summary

The injury rate of Time-Loss injuries for The Currie Cup 2018 was 82 (64 to 100) injuries per 1000 player hours, which is almost identical to the rate of the meta-analysis of international studies, 81 (63 to 105) injuries per 1000 player hours[1], and equates to 1.6 injuries per team per match or 3 injuries per team for every 2 matches played. The average severity of Time-Loss injuries was 31 days lost, and the median severity was 11 days (IQR 5 to 35). The mean and 95% CI Time-Loss injury rates for all teams combined for the 2014 – 2018 tournaments [82 (75 to 88) injuries per 1000 player hours] is very similar to that of the meta-analysis [81 (63 to 105) injuries per 1000 player hours].

Most of the data in The Currie Cup 2018 Premiership Division Competition were similar to the data shown in the meta-analysis [1], illustrating a similarity with international trends. The injury rate of recurrent Time-Loss injuries in The Currie Cup 2018 Premiership Division Competition was significantly lower than that of the meta-analysis. An interesting point to reflect upon is the following. Of the 77 Time-loss injuries in 2018, 8 players (5% of the player pool) contributed to 29 or 37% of all of the 2018 Tournament’s injuries. The potential impact of this finding needs further interrogation and introspection within the Unions to ascertain why certain players are more prone to injury and/or re-injury than others.

Despite having the highest Time-Loss injury rate DHL Western Province had the second lowest average injury severity of 17 days. The median severity of injuries sustained in DHL Western Province was 6 days with 25% of their injuries lasting 5 days or less and only 25% of their injuries lasting longer than 12 days. This means that they had a high number of injuries but that the severity of their injuries was low resulting in them not losing many training and match days for each injury. In contrast, the Cell C Sharks had the lowest Time-Loss injury rate, but the highest average injury severity of 54 days. They had a median injury severity of 13 days with 25% of their injuries lasting less than 9 days and 25% of their injuries lasting longer than 93 days. This means that even though the Cell C Sharks had a low number of injuries, a large number of training and match days were lost due to those injuries. It is important to note that the return-to-play date of an injured player will, to an extent, be influenced by the rehabilitation approach taken by the team. Teams who take a more conservative approach to the rehabilitation of injuries will reflect an increased total injury severity.

When comparing tournament years, DHL Western Province experienced a significant increase in injury rate from 2017 to 2018, while the iCollege Pumas experienced a significant decrease in injury rate from 2017 to 2018. The results of this study highlight the importance of collecting severity data, and not simply injury rates on their own, as although teams may have a low injury rate, injuries of a high severity still represent a burden to the team resulting in a large number of training and match days lost due to injury.

There were three injuries removed from the severity analysis due to the specific nature of these injuries and the large level of inaccuracy in trying to determine their severity. It must however, be acknowledged, that removing an injury of such a nature has a substantial knock-on effect on the injury severity calculations, and the interpretation of the involved data must be performed with this in mind.

An interesting finding in this year’s report was that, while the tackle-related injuries came down, the proportion of injuries sustained in the *Scrum* and *Open play* phases in The Currie Cup 2018 were significantly higher than the average proportion of injuries sustained in those phases in the combined 2014 – 2017 tournaments. *‘Acceleration’* accounted for 60% (n=9) of injuries in the Scrum and 63% (n=12) of injuries in Open play in The Currie Cup 2018. Expanding on the *‘acceleration’* injuries reveals that in the Scrum, 7 of 9 injuries were to the lower limb, of which 5 were muscle strains. In Open play, 10 of the 12 injuries were to the lower limb, of which 5 were muscle strains. This is important to note as muscle strain injuries are preventable through appropriate conditioning and prehabilitation exercise.

When combining all injuries from 2014 – 2018; 58% of all *moderate* and *severe* injuries occurred to the head, knee, ankle, shoulder and AC joint areas. We have recently started a video analysis project to further analyse and describe the mechanisms contributing to these injuries. This will become a focus area over the next few years.

A study from the English Premiership rugby competition showed that Time-Loss injuries hamper team performance [6]. Teams with better injury profiles (lower injury burdens) performed better in the tournament, over a seven-year period [6]. It is interesting to note in 2018 the Cell C Sharks, who finished top of the log had the lowest injury rate. For the past five years the team with the lowest injury rate in The Currie Cup has finished in either first or second position at the end of the competition.

Injury surveillance is the critical first step in the development of injury prevention strategies for a particular surveyed group. After five years of injury surveillance in The Currie Cup competition these data now provide a well powered dataset, and provide a strong evidence-base for developing targeted injury prevention strategies. The next step in this process will be to workshop potential strategies with all key role players involved. Using this dataset as the evidence-base for identifying those key areas that need intervention, this workshop will need to focus on finding practical solutions in an attempt to address the identified areas of concern.

An inherit limitation in this type of study is that the data collection process is entirely dependent on the doctor declaring all medical injuries. The aim of these reports is to describe the injury profile of The Currie Cup Premiership Division competition and subsequently develop strategies to reduce the number of injuries in the tournament. This can only be maximally beneficial if complete and accurate data is collected by all teams involved in the tournament.[Fig f32-2078-516x-31-v31i1a6532][Fig f33-2078-516x-31-v31i1a6532]

**Figure f32-2078-516x-31-v31i1a6532:**
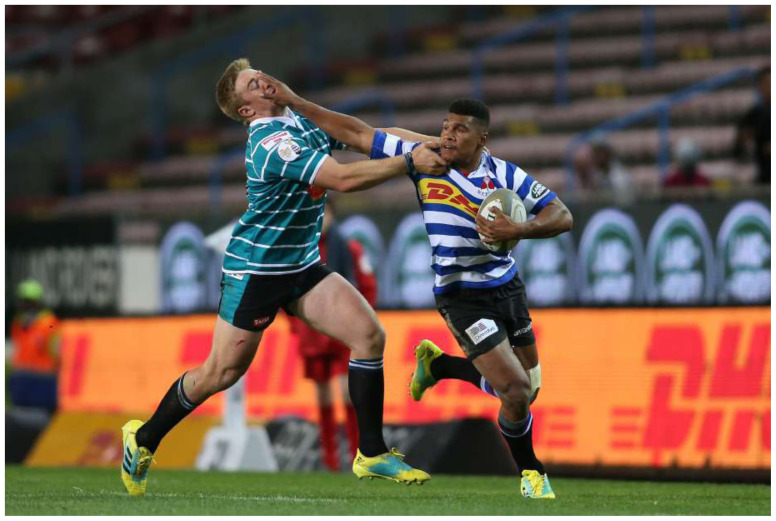


**Figure f33-2078-516x-31-v31i1a6532:**
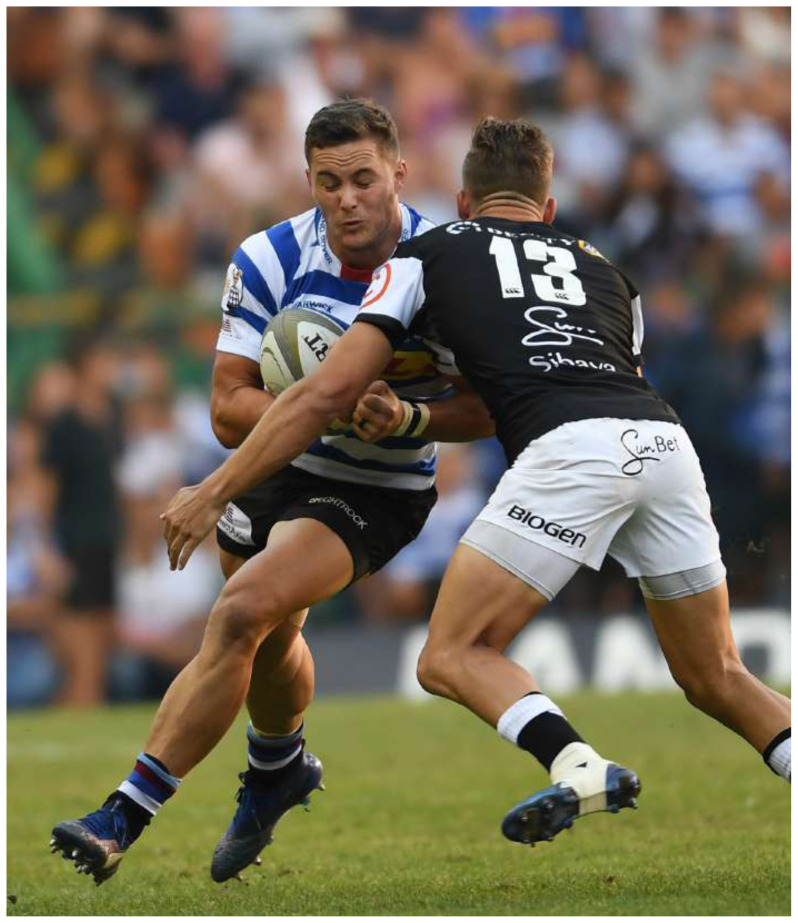


## Figures and Tables

**Figure 1a:**
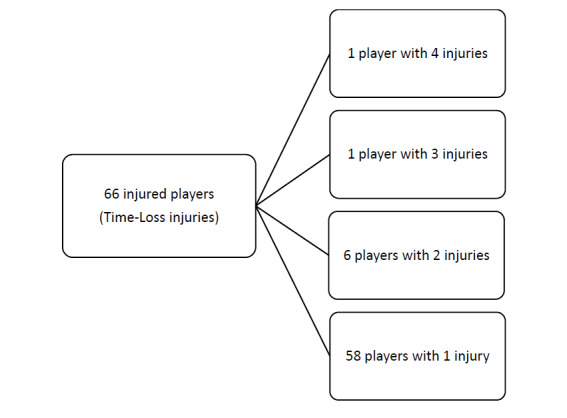
The number of players who experienced Time-Loss injuries during The Currie Cup 2018.

**Figure 1b:**
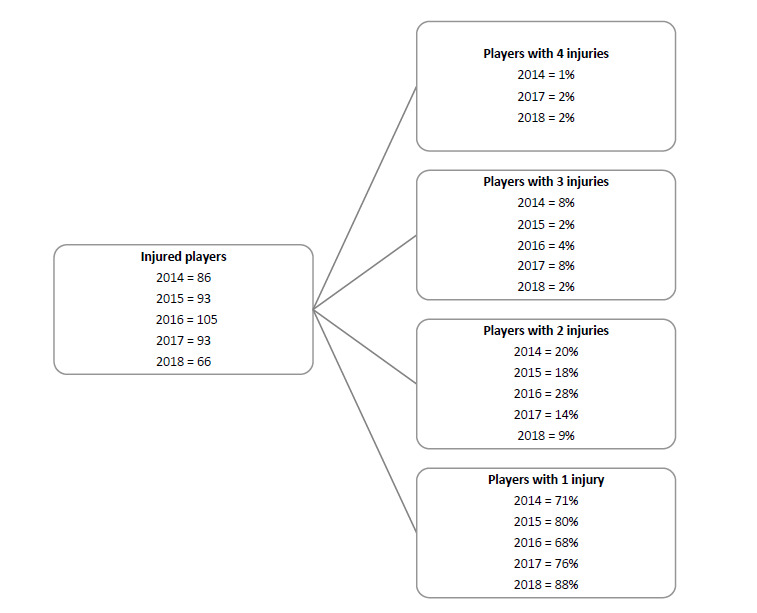
Proportion of players who experienced Time-Loss injuries in The Currie Cup tournaments of 2014, 2015, 2016, 2017 and 2018.

**Figure 2a:**
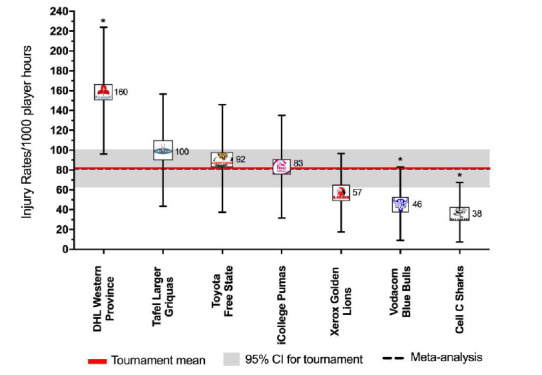
Match Time-Loss injury rates of the seven teams participating in The Currie Cup 2018, including the mean and 95% confidence intervals (CI) for all teams combined. Time-Loss injuries n=77. Asterisk (*) indicates that a team’s injury rate is significantly different to another team in the tournament for that year.

**Figure 2b:**
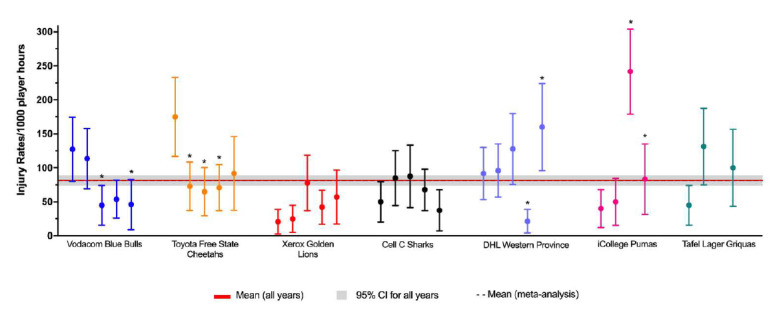
Injury Rate/1000 player hours for Time-Loss injuries experienced by each team in The Currie Cup 2014, 2015, 2016, 2017 and 2018 tournaments. Injury rate is displayed for each team for each year of participation, with from left to right, 2014 being the first point for each team and 2018 being the last point. iCollege Pumas display points for 2015 – 2018 and Tafel Lager Griquas display points for 2015, 2016 and 2018. Asterisk (*) indicates that a team’s injury rate is significantly different to their own injury rate in a previous year.

**Figure 4a:**
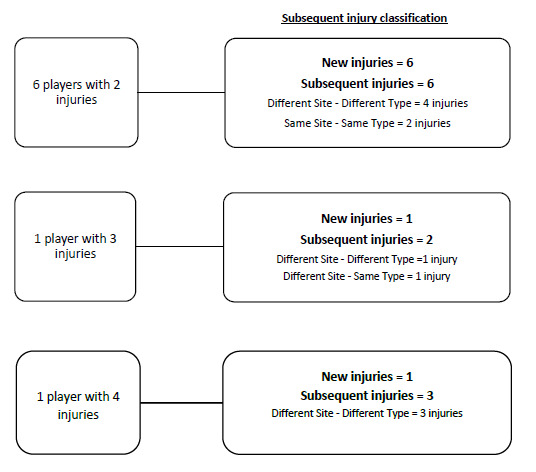
Classification of subsequent Time-Loss injuries sustained during The Currie Cup 2018 (8 players sustained more than one injury; the first injury for each player was recorded as a new injury and the following as subsequent injuries. New injuries n = 8; subsequent injuries n = 11).

**Figure 4b:**
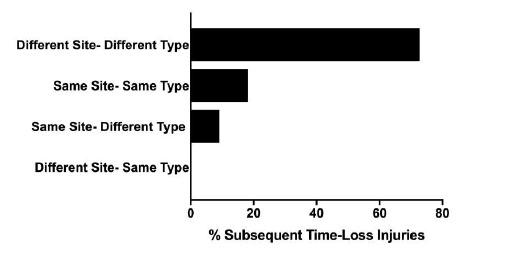
Classification of subsequent Time-Loss injuries for The Currie Cup 2018. Data expressed as a % of subsequent Time-Loss injuries.

**Figure 16a:**
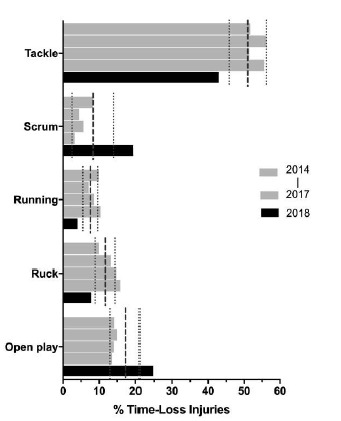
Most common injury events for The Currie Cup 2014 (n=114), 2015 (n=120), 2016 (n=142), 2017 (n=126) and 2018 (n=77) tournaments. Data expressed as % of Time-Loss injuries with mean and 95% CI for each event indicated with dotted lines.

**Figure 16b:**
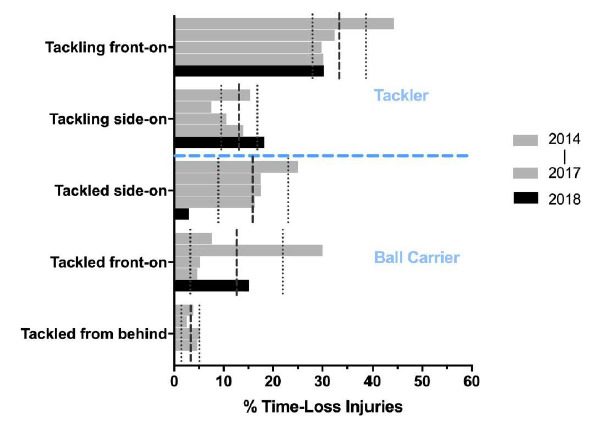
The top five tackle related injury mechanisms of Time-Loss injuries for The Currie Cup 2014 (n=114), 2015 (n=120), 2016 (n=142), 2017 (n=126) and 2018 (n=77) tournaments. Data expressed as % of tackle related injuries, with mean and 95% CI for each mechanism indicated with dotted lines.

**Figure 17 f17-2078-516x-31-v31i1a6532:**
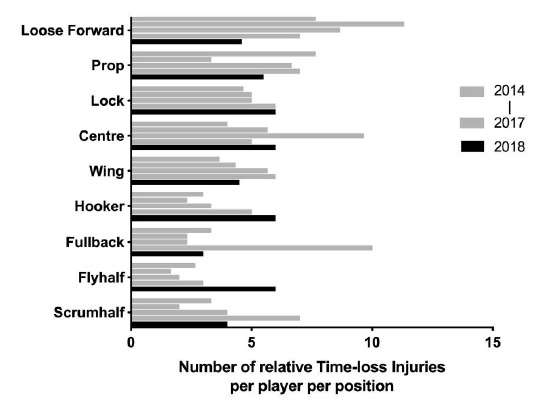
The position of injured players for Time-Loss injuries adjusted for number of players per position for 2014 (n=120), 2015 (n=114), 2016 (n=142), 2017 (n=126), 2018 (n=77).

**Table 1:** Injury rate per team, risk per player, and severity per team for Time-Loss injuries for The Currie Cup 2018.

Team	Injury Rate (95% CI)	Team Injuries/Match	Player match hours/Team injury	Chances of injury per player per match (%)	Number of matches per player before an injury can be expected
Vodacom Blue Bulls	46 (9 to 83)	1.1	21.7	6	16.3
Toyota Free State Cheetahs	92 (38 to 146)	1.8	10.9	12	8.2
Xerox Golden Lions	57 (18 to 97)	1.1	17.5	8	17.5
iCollege Pumas	83 (32 to 135)	1.7	12.0	11	9.0
Cell C Sharks	38 (8 to 68)	0.8	26.3	5	19.7
DHL Western Province	160 (96 to 224)	3.2	6.3	21	4.7
Tafel Lager Griquas	100 (43 to 157)	2.0	10.0	13	7.5

**Table 2:** Severity (days), Injury Burden (days absent/1000 player hours) and Operational Burden (days absent/injury/match) of Time-Loss injuries for each participating team in The Currie Cup 2018.

Team	Team Injuries/match	Team matches/injury	Total Severity	Average Severity	Injury Burden	Operational Injury Burden	Median Severity (IQR)
Vodacom Blue Bulls	0.9	1.1	143	24	1096	21	24 (11 to 36)
Toyota Free State Cheetahs	1.8	0.5	171	16	1430	29	5 (4 to 16)
Xerox Golden Lions	1.1	0.9	273	34	1945	39	14 (9 to 58)
iCollege Pumas**	1.7	0.6	434	54	4503	90	25 (5 to 77)
Cell C Sharks**	0.8	1.3	270	54	2052	41	13 (9 to 93)
DHL Western Province	3.2	0.3	417	17	2780	56	6 (5 to 12)
Tafel Lager Griquas	2.0	0.5	550	46	4583	92	23 (12 to 56)

** Injuries removed from severity calculations.

Pumas n = 2, Sharks n = 1.

**Table 3:** Injury rate, Severity and Burden of the top 5 most common injury types in The Currie Cup 2018.

Injury Type	Injury Rate (95% CI)	Total Severity	Average Severity	Average Burden	Operational Injury burden	Median (IQR)
Central Nervous System	20 (11 to 29)	384	21	427	9	12 (9 to 16)
Sprained Ligament	15 (7 to 23)	510	39	589	12	34 (5 to 61)
Muscle (Rupture/Strain/Tear)	13 (6 to 20)	302	25	327	7	8 (5 to 25)
Haematoma	6 (1 to 12)	37	6	37	1	7 (4 to 8)
Contusion/Bruise	5 (1 to 10)	18	4	4	0.1	4 (3 to 4)

**Table 4:** Injury rate, Severity and Burden of the top 5 most common injury types in The Currie Cup 2018.

Injury Type	Injury Rate (95% CI)	Total Severity	Average Severity	Average Burden	Operational Injury Burden	Median (IQR)
Head	15 (7 to 23)	192	18	206	4	12 (9 to 16)
Shoulder	9 (3 to 14)	298	38	298	6	18 (10 to 33)
Knee	9 (3 to 14)	307	44	351	7	35 (19 to 70)
Ankle	7 (2 to 13)	458	65	458	9	89 (32 to 92)
Anterior thigh	6 (1 to 12)	36	6	393	8	5 (5 to 7)

**Table 5:** Ball-in-play time (mins) and injury incidence/1000 BIP time hours of Time-Loss injuries for The Currie Cup 2014 (n=114), 2015 (n=120), 2016 (n=142), 2017 (n=126) and 2018 (n=77) tournaments.

	Average ball-in-play time (range) per match (mins)	Number of matches	Number of teams	Total ball-in-play time (mins)	Averaged total ball-in-play time per team	Injuries per team per ball-in-play time per minute	Injuries per team per theoretical 80 minute match	Injuries per team per actual 80 minute match	Time-loss Injury incidence/1000 ball-in-play hours (95% C.I)	Time-loss injury incidence/1000 player hours (95% C.I)
2014	32 (27 – 38)	33	6	2135	5h 55 mins	0.06	4.5	1.5	225 (185 – 265)	91 (75 – 107)
2015	30 (22 – 35)	43	8	2603	5h 25 mins	0.04	3.5	1.4	175 (143 – 207)	66 (54 – 78)
2016	32 (26 – 37)	39	9	2468	4h 34 mins	0.06	4.6	1.8	230 (192 – 268)	91 (76 – 106)
2017	35 (29 – 42)	39	6	2696	7h 29 mins	0.05	3.7	1.6	188 (154 – 220)	81 (67 – 95)
2018	32 (28 – 42)	24	7	1551	3h 41 mins	0.05	3.9	1.0	199 (154 – 243)	82 (64 – 100)
**Combined average**	**32 (26 – 39)**	**36**	**7**	**2291**	**5h 25 mins**	**0.05**	**4.0**	**1.4**	**202 (186 – 219)**	**82 (75 – 88)**

## Footnotes

5 Orchard, J. *Orchard Sorts Injury Classification System (OSICS)* 2019; Available from: https://www.johnorchard.com/osics-downloads.html.

## References

[1] Williams S A meta-analysis of injuries in senior men's professional rugby union Sports Med 2013 43 10 1043 55 23839770 10.1007/s40279-013-0078-1

[2] West S England Professional Rugby Injury Surveillance Project: 2017/18 2018 Rugby Football Union (RFU) 1 60

[3] Fuller CW Consensus statement on injury definitions and data collection procedures for studies of injuries in rugby union Br J Sports Med 2007 41 5 328 31 17452684 10.1136/bjsm.2006.033282 PMC2659070

[4] Schenker N Gentleman JF On Judging the Significance of Differences by Examining the Overlap Between Confidence Intervals The American Statistician 2001 55 3 182 186

[5] Orchard J John Orchard’s Sports Injury Site - OSICS Download 2019 [cited 2019 01, 06]; Available from: https://www.johnorchard.com/osics-downloads.html

[6] Williams S Time loss injuries compromise team success in Elite Rugby Union: a 7-year prospective study Br J Sports Med 2015 0 1 6 10.1136/bjsports-2015-094798 26552415

